# Enabling Research and Clinical Use of Patient-Generated Health Data (the mindLAMP Platform): Digital Phenotyping Study

**DOI:** 10.2196/30557

**Published:** 2022-01-07

**Authors:** Aditya Vaidyam, John Halamka, John Torous

**Affiliations:** 1 Beth Israel Deaconess Medical Center Boston, MA United States; 2 Mayo Clinic Rochester, MN United States

**Keywords:** digital phenotyping, mHealth, apps, FHIR, digital health, health data, patient-generated health data, mobile health, smartphones, wearables, mobile apps, mental health, mobile phone

## Abstract

**Background:**

There is a growing need for the integration of patient-generated health data (PGHD) into research and clinical care to enable personalized, preventive, and interactive care, but technical and organizational challenges, such as the lack of standards and easy-to-use tools, preclude the effective use of PGHD generated from consumer devices, such as smartphones and wearables.

**Objective:**

This study outlines how we used mobile apps and semantic web standards such as HTTP 2.0, Representational State Transfer, JSON (JavaScript Object Notation), JSON Schema, Transport Layer Security (version 1.3), Advanced Encryption Standard-256, OpenAPI, HTML5, and Vega, in conjunction with patient and provider feedback to completely update a previous version of mindLAMP.

**Methods:**

The Learn, Assess, Manage, and Prevent (LAMP) platform addresses the abovementioned challenges in enhancing clinical insight by supporting research, data analysis, and implementation efforts around PGHD as an open-source solution with freely accessible and shared code.

**Results:**

With a simplified programming interface and novel data representation that captures additional metadata, the LAMP platform enables interoperability with existing Fast Healthcare Interoperability Resources–based health care systems as well as consumer wearables and services such as Apple HealthKit and Google Fit. The companion Cortex data analysis and machine learning toolkit offer robust support for artificial intelligence, behavioral feature extraction, interactive visualizations, and high-performance data processing through parallelization and vectorization techniques.

**Conclusions:**

The LAMP platform incorporates feedback from patients and clinicians alongside a standards-based approach to address these needs and functions across a wide range of use cases through its customizable and flexible components. These range from simple survey-based research to international consortiums capturing multimodal data to simple delivery of mindfulness exercises through personalized, just-in-time adaptive interventions.

## Introduction

### Background

The medical field today is transitioning toward integrating patient-generated health data (PGHD) into clinical care to increase shared decision-making, coordination of care, patient safety, and clinical outcomes [1]. PGHD are central to this mission and are defined as data recorded or created by the patient or caregivers used to address health concerns. Examples include a longitudinal view of symptoms of patient status between clinic visits captured via an app or information related to daily adherence to treatment plans [1]. This may include a daily step count captured from a smartphone, medication surveys administered on the smartphone, and sleep quality data measured via a wearable sensor. Given the ability of smartphones and wearables to collect a myriad of continuous multimodal data relevant to care, such as heart rate, sleep, steps, and more, tools and systems to harness and use this vast amount of automatically generated PGHD are a health care priority. One challenge that remains toward this goal is the lack of technical infrastructure and organizational capability to handle the intake of accurate and valid PGHD from patient-owned consumer devices. Such standards and tools supporting the effective and compatible integration of PGHD are needed to enhance clinical insight and support research and data analysis before PGHD can actually impact routine care.

### Digital Phenotyping+

The need for apps that can not only capture but also use and integrate PGHD is clear. The use of commercially available wearable technology for the acquisition of PGHD has seen a recent uptick owing to the effects of the COVID-19 pandemic and growing demand for telehealth services [2,3]. Today, over 80% of Americans own a smartphone device [4] and over 20% own a wearable device [5], reinforcing the potential of PGHD to improve clinical outcomes. For example, the Apple Watch today retails at US $350 with onboard nonmedical grade electrocardiography and oxygen saturation sensors that continuously measure and record data. Modern smartphones are also equipped with numerous sensors that generate a high volume of potentially clinically significant PGHD that could enable a better real-time understanding of cognition, mobility, sociability, and more through techniques, such as digital phenotyping and ecologic momentary assessment.

Digital phenotyping [6] is the construction of an individual-level phenotype using data collected from smartphones or wearable devices actively via user interaction (eg, surveys), or passively without user input (eg, sensors, such as an accelerometer). Although there are many digital phenotyping tools and systems used in health care and research contexts, a recent review identified nearly 50 of them [7], and few offer an integrated and standardized approach to analyze and respond to clinically actionable patient-generated data. Existing tools are primarily closed systems or consist of only a single app with little flexibility or customizability [7]. In a recent review, 85% of existing solutions supported active and passive sensing but only 33% supported clinical assessment, 30% supported predictive modeling of patient data, and 24% supported app-delivered interventions [7]. Furthermore, only 35% of the existing solutions showed a patient- or clinician-facing user interface [7]. However, the combination of all these features is necessary to meet the diverse needs of research and care delivery. A search outside the research literature and instead, across mental health smartphone apps in commercial marketplaces reported that only 1.1% supported sensors [8], suggesting that many research tools do not translate into accessible tools for patient or clinician use. Although diverse functionality and innovation continue to exist across the entire app space, we have argued that there is a need for multiple uses of the same app, instead of using multiple apps in a fragmented manner, toward better supporting clinical research, integration, and implementation [9].

### Challenges in Integration

Creating PGHD tools that use sensor and digital phenotyping tools in a more patient- and health system–centric manner is a common goal, but it remains challenging to achieve. Despite the prevalence of existing electronic medical record standards and tools, a 2019 review on the integration of PGHD into clinical practice, “integration [...] was extremely limited, and decision support capabilities were for the most part basic” [10]. The most widely adopted medical record standards initiative that can be used to link PGHD to medical records is Fast Healthcare Interoperability Resources (FHIR), led by the Health Level 7 organization [11], which is now adopted by many major health care systems and industry partners, including Apple, Google, Amazon, Microsoft, and others [12]. Its companion projects SMART (Specific, Measurable, Achievable, Realistic, and Timely) [13] and SMART Markers [14] build on FHIR and enable integration of third-party modules into medical record systems, including patient mobile devices and sensors.

However, the FHIR ecosystem alone does not address a number of concerns specific to the integration of PGHD into clinical systems. FHIR and the current data interoperability standard (United States Core Data for Interoperability) [15] were not developed for continuous high-velocity data, and its implementation in the health care ecosystem today is primarily read-only, although its data gathering and write-back ability continues to evolve. For example, the FHIR core does not allow for semantic equivalence of data that can be used to automate data matching or harmonization. As a result, it is not possible to work with both cognitive assessment scores and mobility or sociability metrics using the same analysis pipeline. This increases the effort required and time taken to work with PGHD, as clinicians or researchers must first preprocess data of different semantic types individually before being able to work with a data set as a whole. Although R4 extensions, such as the mCODE core cancer model [16] are becoming a new way to expand FHIR’s supported vocabulary, they are still early in evolution and adoption. Today, the inability to standardize terminologies across interconnected systems, such as through a *data dictionary*, impedes effective export and analysis of different types of data from different data sources using the FHIR ecosystem [17,18]. These challenges preclude the adoption of FHIR as a PGHD-first standard for clinical and research use cases.

Thus, there remains a need for a flexible, interoperable, and extensible platform that enables the effective use of PGHD through widely accepted standards for both clinical and research needs. In this paper, we present a potential solution for the robust and effective acquisition and integration of PGHD into research and clinical care with tangible examples and open-source code.

## Methods

### Overview

To integrate PGHD into research and clinical care needs, our team has designed and developed the Learn, Assess, Manage, and Prevent (LAMP) platform that encompasses a robust set of protocols, standards, tools, and apps. Our team initially developed the mindLAMP smartphone app [19] as part of the initial version of the LAMP platform. In this paper, we review a rearchitected and redeveloped platform comprising new frontend, backend, and analysis components to support PGHD, patient-centric care, and actionable digital phenotyping. This new platform, distinct from its predecessor, includes features such as customizable and schedulable activities, sensor data collection and analysis, messaging support with the care team, and more, available across modern web browsers and smartphone operating systems. The design and development of the platform was approached from both a patient- and clinician-focused approach as well as a semantic standards–based approach.

### Patient- and Clinician-Led Design

**Figure 1:**
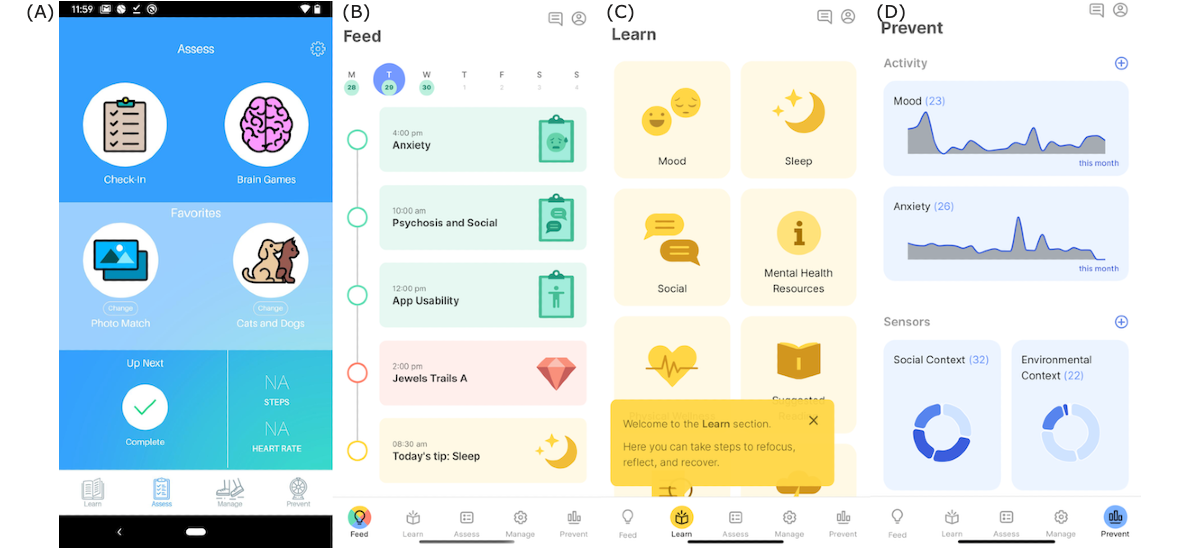
(A) The home screen interface of the original (version 1 with red border) mindLAMP app; (B) the new and improved home screen interface of the (version 2 with green border) mindLAMP app that incorporates multiple activities and schedules into a heads-up tab called the Feed; (C) the Learn, Assess, Manage, and Prevent tabs group embedded activities together with helpful tooltips and icons, with new activity types (eg, tips, meditation); (D) returning data and insight to users was considered a priority in the redesigned user interface and thus more advanced charting tools were integrated into the Prevent tab, accessible to both patients and clinicians.

The LAMP platform was designed and developed with continuous feedback from patients with serious mental illnesses and clinicians. Through a patient advisory panel, focus groups [20-22], clinical use, and feedback from a global consortium of users, mindLAMP has been co-designed iteratively with updates reflecting expanding ideas for its role. User input informed the adaptability, flexibility, and customizability of the LAMP platform, which resulted in a new user interface compared with the previous version, as shown in Figure 1. We established a formal system to enable anyone to suggest improvements, report bugs, and assess new features to ensure that all could partake in the iterative design rounds. This process also influenced aspects of the user experience, such as making mindLAMP available in multiple languages (English, Spanish, and Hindi) and designing to ensure easy addition of more. Key examples of patient feedback and the design outcomes they influenced are provided in Table 1.

**Table 1:** Selected examples of patient feedback driving significant changes in the user experience and overall architecture of the Learn, Assess, Manage, and Prevent platform. Semantic (technical) standards–based approach.

Sample patient feedback	Outcome
“Apps like Facebook or Amazon are clear where I am lost in a sea of people or items and that is generally accepted, but with apps in the health space, who is involved, which institution is involved, level of comfort with the individuals and what data is collected, all of these factors are carefully calculated when I make a decision to join a study like this—an establishment of trust is crucial. Apps that track people incur a level of suspicion that changes between people, from none at all to a lot, perhaps depending on level of illness.”	The Learn, Assess, Manage, and Prevent platform was rebuilt around an open-source collaborative environment supported by the consortium; all development and data handling processes are disclosed in the privacy policy, and significant backend changes were made to patient data equity and ownership.
“Yeah, because I don’t see any apps out there these days that help people with psychosis and when they’re getting sicker. It just seems...they just don’t help with certain things. This gives you control to go get help if somebody needs it. It’s like, the good thing about this app is, it’s getting the right information and it’s sending you somewhere, it’s almost as if you could go to the therapist with this information! You don’t want an app that’s just one sided [and siloed off from the therapist or delivery of care].”	Additional types of activities were added to the mindLAMP app, including tips (Figure 2), meditation, and other informational and management tools; each of these activities captures metadata during patient use that can be interpreted and incorporated into a clinical encounter.
“mindLAMP is a tool for me to get better: I want to know if I’m making progress and when, what am I deficient in, how am I deficient, and how to improve on it; that is, as a metrics-driven person.”	The smaller heads-up summaries originally found in the first version of the mindLAMP app (Figure 2) were updated and expanded into an entire tab (Figure 2), providing more insight and customization into patient data.

**Figure 2:**
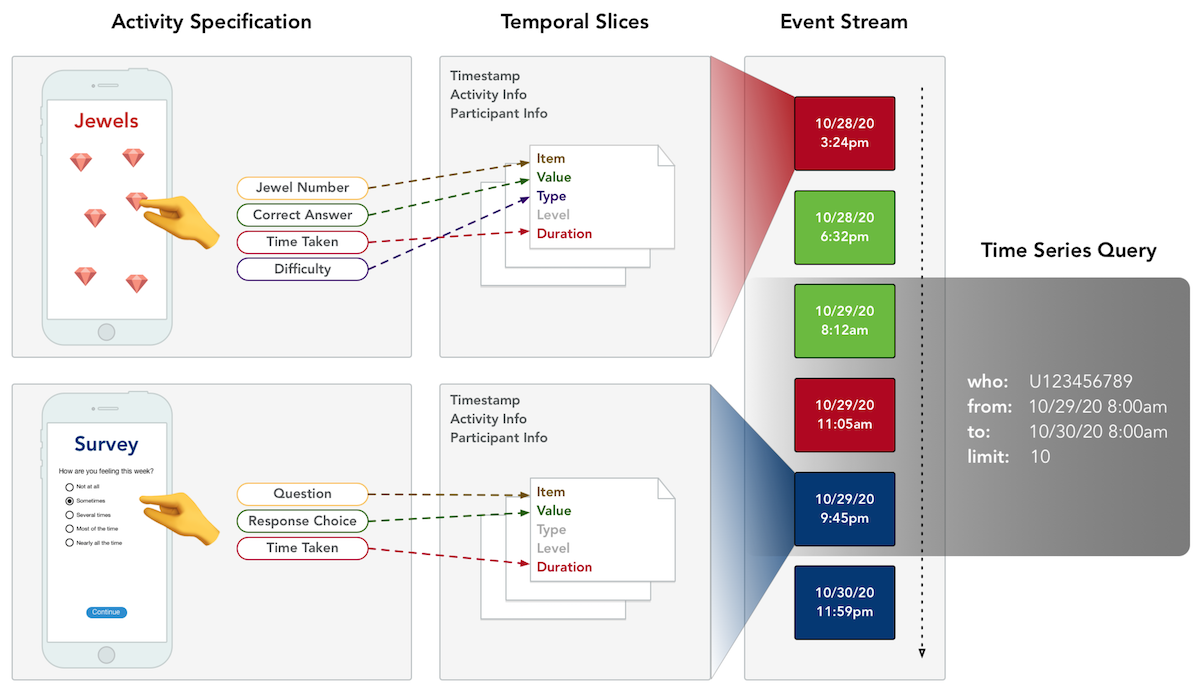
Flow of the data collection process from native app to backend: (1) an activity specification describes the types of interactive elements available in the mindLAMP app, along with their possible configuration parameters; (2) when participants interact with a configured and scheduled activity (such as a mood survey based on the survey specification), all metadata and data from the interaction session is integrated into a single unit of patient-generated health data called events; (3) events are then submitted to the backend in real time as part of a continuously generated stream of patient-generated health data; and (4) clinicians and researchers are able to perform continuously updating queries on the data with their desired parameters.

In addition to a patient- and clinician-centric design approach, the LAMP platform was also architected with a semantic standards–based approach, considering technical best practices for future proofing and security or compliance across health care systems. The open standards listed in Table 2 were chosen specifically to foster an open ecosystem around the platform. For example, the platform’s programming interface adopts a *repository* model to store and configure patient-facing instruments, each with its own embedded user interface. These embedded user interfaces were developed using common and widely adopted web standards indicated in Table 2 (HTML5.0, Cascading Style Sheets 3.0, and ECMAScript 6). As a patient begins an interaction session, this embedded code is securely sandboxed by the mindLAMP user interface both within the smartphone app and the patient-facing web dashboard. In addition to providing a standard schematic of all structured documents encountered and processed in the LAMP platform using OpenAPI [23], the JSON Schema [24] data markup standard is used to provide developers of these interactive patient-facing instrument configurability and extensibility. With little required skill or upfront effort, developers can use the platform’s software development kit to create instruments with completely customizable user experiences that are then tuned and customized by clinicians for individual patients or by research coordinators for studies spanning many patients.

**Table 2:** Adopted semantic web standards, their use rationale, and implementation details.

Standard	Description	Use	Reason chosen
HTTP 2 [22]	Ubiquitous web standard that declares and defines the semantics of client-server communication with a rich and readily available debugging and implementation toolset and ecosystem not available for custom binary protocols	Implemented by core programming libraries and the backend	In contrast to TCP^a^-based binary data protocols requiring specialized tooling to access and work with data, almost all systems and tools are able to interact with web standards through the HTTP protocol.
REST^b^ [25]	Ubiquitous lightweight HTTP-based web standard that defines systems logically through accessibility and manipulation of remote resources instead of invocation of remote functions	Implemented by core programming libraries and the backend	In contrast to a custom implementation of remote function invocation that would require custom programming libraries to interface with, most web systems are able to interact with REST-based resources in a logical manner. In the absence of developer knowledge or pre-existing tools, it remains possible to communicate with RESTful systems.
JSON^c^ [26]	Ubiquitous web standard that supports structured (as opposed to tabular, ie, CSV files) formatting and markup of data using strict data types	Implemented across all components in the platform	In contrast to encoded binary data formats requiring specialized tooling to interpret and work with data, most programming environments support the JSON standard.
TLS^d^ version 1.3 [27]	Ubiquitous web standard that enables encryption of data in transit between client and server	Implemented by core modules and programming libraries, used by all components in the platform	No alternative
AES-256^e^ [28]	Ubiquitous cryptographic standard that enables encryption of data at REST (on disk) by a database	Implemented by the backend and mandated by the backend and deployment configuration for the database within which data shall be stored	No alternative
JSON Schema [24]	Web standard that describes JSON-encoded data and metadata through ahead-of-time specification of a universally agreed upon schematic, as opposed to inline schema provided only at runtime	Implemented by the backend and used by the frontend	Although binary protocols require a predetermined strict schema to format the data, JSON does not. JSON Schema provides ahead-of-time resolution of the contents of a data payload and can be used to validate and harmonize data as well.
OpenAPI [23]	Web standard that describes REST-based web services and metadata through ahead-of-time specification of a universally agreed upon schematic	Implemented by the backend and core programming libraries and used by the frontend	In contrast to writing programming libraries and testing or validation tools, the generation of these tools and packages by the OpenAPI ecosystem increases productivity.
HTML5 [29]	Ubiquitous web standard that makes it possible to securely embed custom user interfaces backing patient-facing activities	Implemented by the frontend and all patient-facing activities, with wide support for CSS3^f^ and JavaScript 2016 (ES6^g^)	No alternative
Vega [30]	Visualization grammar standard that encodes charts and graphs as JSON documents that are then rendered and viewed interactively by apps	Implemented using HTML5. Implemented by the frontend and Cortex analysis code	In contrast to static images and handwritten analysis code, the ability to declaratively generate interactive real-time charts through an embedded query reduces data science and clinician effort and fatigue.

^a^TCP: transmission control protocol.

^b^REST: Representational State Transfer.

^c^JSON: JavaScript Object Notation.

^d^TLS: Transport Layer Security.

^e^AES-256: Advanced Encryption Standard.

^f^CSS: Cascading Style Sheets.

^g^ES: ECMAScript.

### LAMP Platform

**Figure 3:**
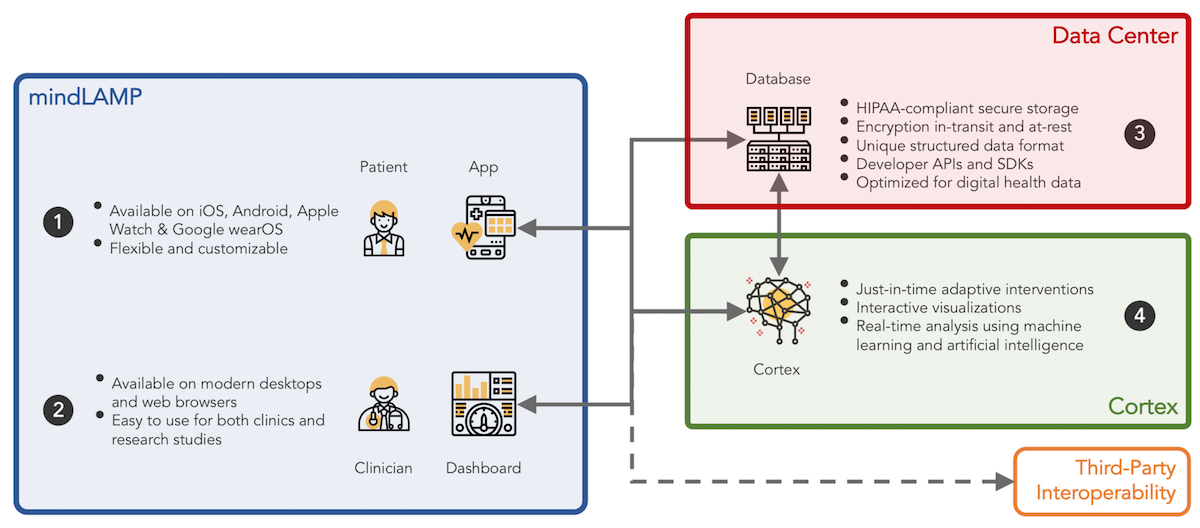
The Learn, Assess, Manage, and Prevent platform consists of three major components: (1) mindLAMP, the patient- and clinician-facing app and web dashboard; (2) Data center, providing secure storage and access to data; and (3) Cortex, the data analysis toolkit that enables adaptive interventions and interactive visualizations. API: application programming interface; HIPAA: Health Insurance Portability and Accountability Act; iOS: iPhone operating system; SDK: software development kit.

**Figure 4:**
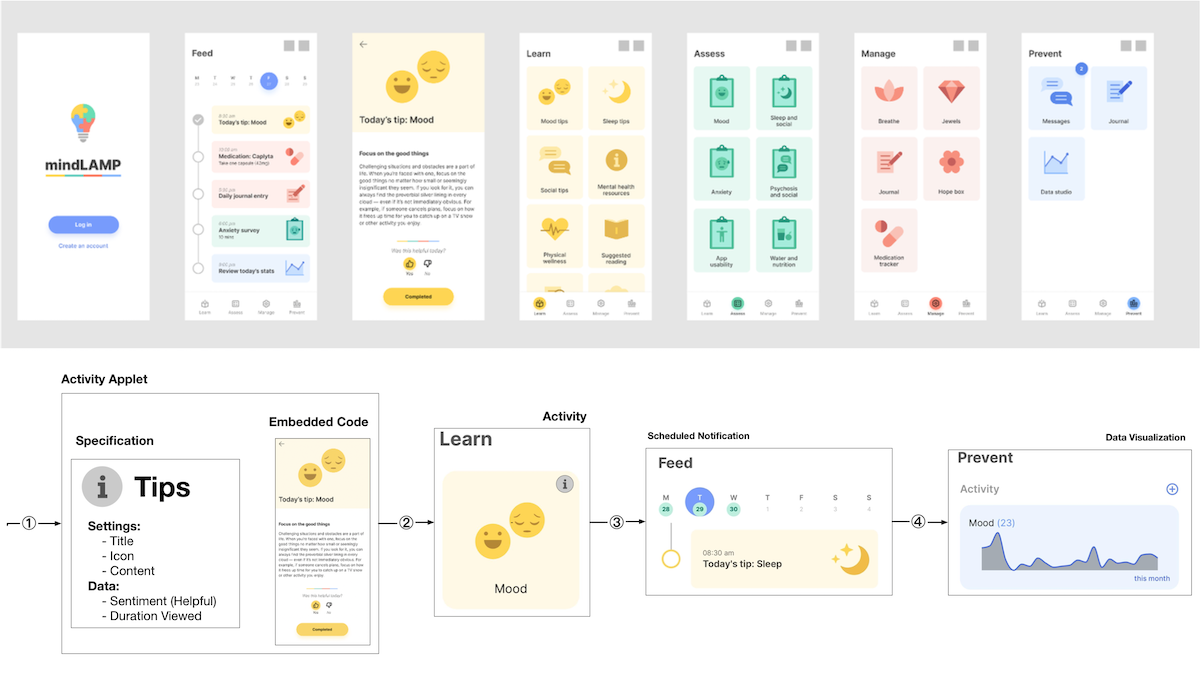
Screenshots of the home screen as viewed by a patient in the mindLAMP app on a smartphone device; system administrators install applets such as Survey, Breathe, or Tips that clinicians and researchers are able to configure and schedule so that the participants interact with the app and produce data and metadata.

The LAMP platform is a customizable clinical care management and neuropsychiatric research platform designed around PGHD, as detailed in Figure 3. It comprises numerous essential features, such as customizable clinician-defined activities (eg, surveys, breathing exercises, journaling, and cognitive tests), collection and analysis of mobile and wearable sensor data, push notification scheduling, care team–centric conversations, just-in-time adaptive interventions, prebuilt featurization, visualization, or analysis pipelines, and a companion integrated development environment (IDE). The LAMP platform is available for use across any modern desktop web browser as well as recent versions of iPhone operating system and Android through the mindLAMP app available on the Apple and Google app stores, as shown in Figure 4. The backend is deployable using enterprise-standard orchestration tools (Docker and Kubernetes [31]) and has already been deployed across several health care systems and is used today by patients, clinicians, and researchers. The companion Cortex data analysis toolkit integrates tightly across the platform to provide a unified processing pipeline for secondary active and passive data features (measurable behavioral characteristics extracted from raw data), interactive visualizations, and the generation of targeted and automated adaptive interventions. The IDE is bundled with support for the widely adopted Python, R, and JavaScript programming languages and built atop Jupyter Notebooks and Visual Studio Code for collaborative data analysis.

The LAMP platform is designed to be customizable to fit a wide range of use cases and requirements, eliminating the need for multiple apps hosting only a set of fixed, immutable content as well as the research concern of proprietary data formats and closed-source commercial analysis software. It also securely enables data interoperability and extensibility, avoiding the issue of *data silos* without external access of collected data for clinicians or patients. It integrates into existing hospital organization structure and is not limited to the *sandbox* on an individual’s smartphone, allowing the caregivers and patients to coexist on the same platform. These features combined allow the LAMP platform to engage the care team through interactive clinical decision support with adaptive responses to incoming PGHD. Where a self-contained app must focus on solving individual problems for specific stakeholders, the LAMP platform focuses on broader challenges around linking people with data and data to teams of interconnected stakeholders, from patients and clinicians and family members and the care team to administrators and research coordinators. For these reasons, the LAMP platform supports *digital phenotyping+*, the plus symbol indicates the ability to return and share PGHD with the individuals from which it is collected in a secure and ethical manner and the ability to integrate that data into a machine learning pipeline or other clinical decision support algorithms.

## Results

### Overview

The rearchitected LAMP platform addresses the integration of PGHD into existing systems through a simplified extensible programming interface (application programming interface [API]) and an internal data representation that interlinks raw data and metadata with descriptive schema. The Cortex data analysis toolkit obviates the need for custom preprocessing or harmonization of disparate sources of data and removes barriers between the real-time collection of PGHD and subsequent featurization, analysis, or visualization. We present the process results and examples below but do not offer a hypothesis in line with other papers exploring informatics systems created for use in clinical care and research.

### Integration to Existing Systems

To enable robust data analysis, adaptive interventions, and interoperability with a broad range of health care systems and services, the platform’s data repository and programming interface are based upon a concise semantic FHIR-compatible API. The platform’s API provides both predefined and pluggable schematics for patient-facing instruments, such as surveys and cognitive tests as well as for mobile and wearable sensors. The platform’s backend validates and harmonizes patient data upon receipt, retaining lossless FHIR compatibility in the process. The platform provides a facility to query and transform data into FHIR-compatible *bundle* and *resource* types, in addition to other domain-specific tabular or structured data formats. Table 2 lists the clinical, regulatory, and software standards implemented and supported by the LAMP platform.

**Figure 5:**
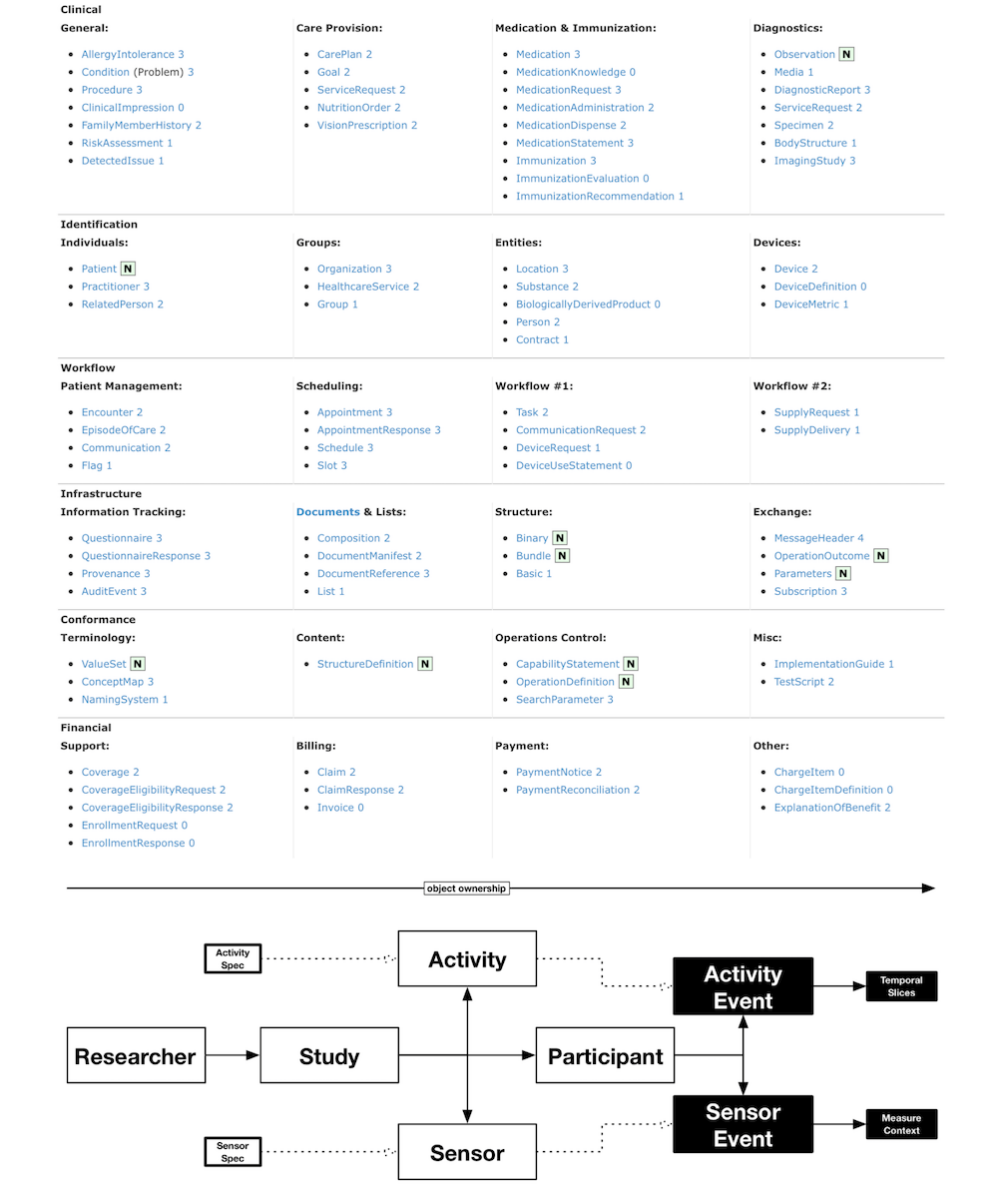
The structured list of all supported Fast Healthcare Interoperability Resources core resources; the structured list of all Learn, Assess, Manage, and Prevent platform resources.

The LAMP platform’s internal data representation provides a simplified abstraction around PGHD in comparison with FHIR. Fundamentally, FHIR adapts a message and document-based exchange programming interface atop the representational exchange state transfer web standard protocol. The FHIR data structures (Figure 5) consist of over 90 *modules* for clinical use, insurance, billing, and other use based on the concept of *resources*, with each resource containing some raw data, metadata, a schema identifier, and a human-readable representation of the raw data. The schema identifier is used to reference how the data should be interpreted by a compatible system or machine. As the raw data contained within 2 resources of the same schema type may differ (eg, the use of the *observation* data type to represent both blood pressure and depression assessments results), data processing cannot be standardized across different data types. By organizing and accessing PGHD separately from generalized repositories of data, such as electronic health record systems using the FHIR API, common and shared analysis methods and processing tools that are standardized across such various data types can be used by clinicians and researchers.

The LAMP platform declares only 12 core PGHD-centric resources (Figure 5) that remain focused on clinical and research use. The *Researcher* and *Study* resource types group together sets of *participants* as well as the *activities* and *sensors* they are able to interact with or collect data from. Upon data collection, *ActivityEvent* and *SensorEvent* describe and link the recorded data to its metadata and any specific customized parameters. The *Credential* resource provides security access controls to any of the aforementioned resources, and the *Tag* resource provides support for integration, extensibility, and backward compatibility. The semantic context of any recorded data and metadata is described by *ActivitySpec*, *SensorSpec*, and *TagSpec*.

### Internal Data Representation

Understanding the need for seamless integration into existing health care systems, software, and services, the platform exposes its internal data representation through the LAMP protocol, a programming interface that enables integration with third-party services. For example, integration with Google Fit wearable devices that also implement this same *push-based model*, is possible by signing up on the Fitbit developer portal and connecting the data output of the Fitbit programming interface to the data input in the programming interface provided by the LAMP platform. In another example of seamless integration, clinicians and researchers can use automated scripts to synchronize data between the mindLAMP app and their existing record-keeping systems. Data can be proactively fetched and stored securely, and users of the platform are notified of any or all data from a particular patient using *subscriptions*, regardless of whether the data were generated by the mindLAMP app or a third-party data source.

The extensibility and flexibility of patient-facing instruments in the LAMP platform rely on the unique data structure and functionality provided by the LAMP protocol as shown in Figure 2. Each activity with which patients are able to interact is defined and encapsulated in an *activity specification* that contains the program code written using web-compatible standards, along with descriptors of the required input configuration and output data. When a patient begins an interactive session with any activity, session-wide metadata regarding *who*, *what*, and *when* are recorded. Each tap of the screen within the activity is then automatically validated and converted into a standardized data format called a *temporal slice*. When the user completes the interactive session, all the *temporal slices* are packaged into chronologically ordered events indexed under the patient’s identifier as a stream of continuously generated data. The data analyst is then able to query these data at any desired temporal resolution (eg, 1 millisecond, 1 day, and 1 year) and filter by the type of activity (eg, mood survey, anxiety survey, trails-making test, and meditation). The query can be mutated using transformation logic executed by the backend and subscribed such that newly uploaded data matching the query is reported in real time to the data analyst. This query framework can be used to better understand how participants use and engage with the activities available to them as part of the study, for example, by extracting a real-time metric of duration spent meditating in the app per participant.

As depicted in Figure 4, the flow of activity specifications to configured activities to their generated PGHD facilitates patient interaction with any kind of interactive web media, from static text for tips, to video content for learning modules, or audio content for breathing exercises. Instruments and their data can be monitored and maintained organization-wide for compliance and conformance. Multimedia Appendix 1 lists the sources of active and passive data currently available within the mindLAMP app and their data sources and types. This novel data organization and structure supported by the platform enables unification and harmonization of these different data types, with both backward compatibility to data types from legacy systems and future compatibility for data types for systems that are not yet available.

### Data Analysis With Cortex

**Figure 6:**
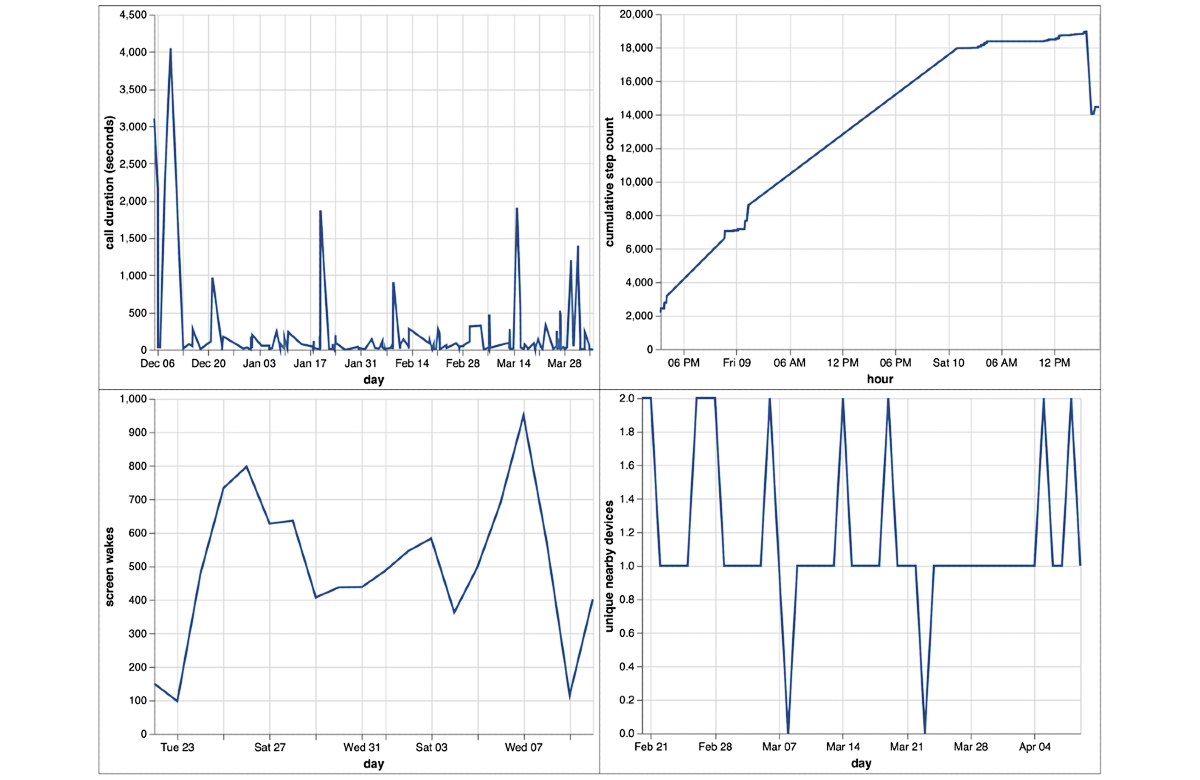
Samples of data from selected sensors in the mindLAMP app for a sample patient. Total duration (in seconds) spent in calls per day; cumulative number of steps taken per hour during a 24-hour rolling window; number of times the device’s screen was turned on per day; number of unique nearby devices (Wi-Fi or Bluetooth) encountered per day.

The same pipeline operates on both active and passive data, unifying the conceptual model for PGHD processing and obviating the need for individual analyses tied to custom code for specific sensor types across various devices. Sensor data are therefore subject to additional harmonization to account for the various differences in functionality and recording between Apple and Android devices. For example, accelerometer measurements taken on Apple devices are measured in units of gravity (G) with a frame of reference experiencing −1 G in the downward-facing axis, whereas measurements on Android are measured in meters per second square (m/s^2^) without a frame of reference provided. As the platform automatically applies this harmonization step, the data analysis code does not require an intrinsic understanding of the source of the data. Samples of sensor data after harmonization are shown in Figure 6. Furthermore, in addition to raw sensors on smartphones or wearable devices, processed Apple HealthKit and Google Fit sensor data, such as activity recognition or heart rate variability, are available to the LAMP platform.

**Figure 7:**
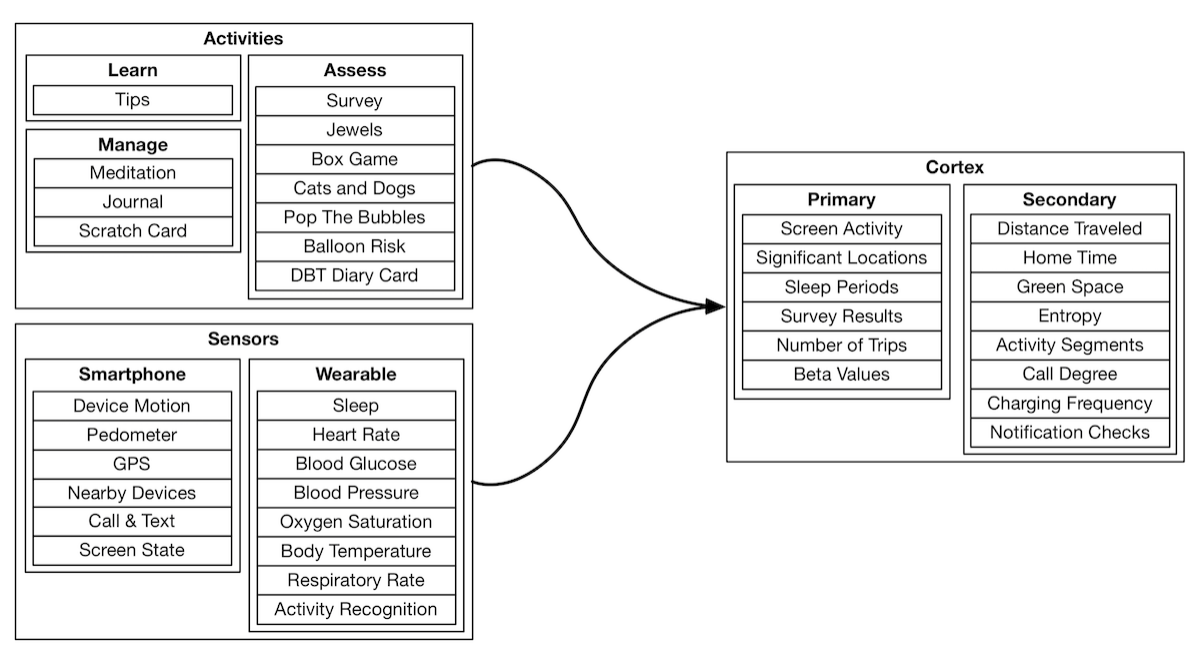
A visual representation of the various categories of activity and sensor data type features using standardized functions as part of the Cortex data analysis toolkit; shown as part of Cortex is the distinction between the primary and secondary feature types, where secondary features are composed of primary features as opposed to raw patient-generated health data. Availability of wearable sensors depends on the device type used and supported application programming interface; Apple Watch (HealthKit) sensors are shown here. DBT: dialectical behavioral therapy.

**Figure 8:**
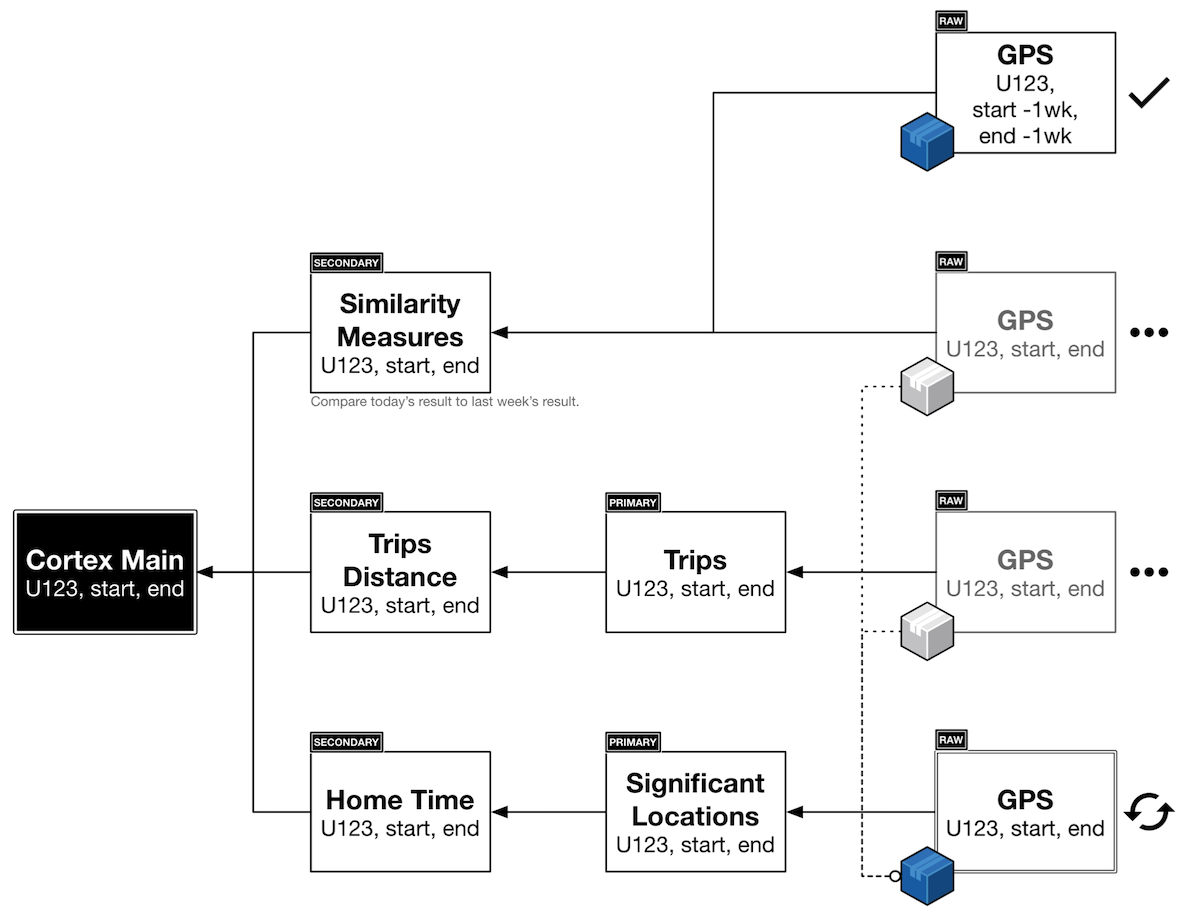
A sample execution plan for Cortex around an example using only geolocation data (1) the clinician or researcher creates an aggregate operation; (2) Cortex transparently interposes the correct feature layers by creating a dependency graph of data and executes each atomic operation (ie, independent of external variables) in the order it computes to be most efficient; (3) any raw sensor data are transparently cached during execution; and (4) as multiple operations require the same raw sensor data, Cortex blocks their execution until the cached data becomes available, to avoid duplicate downloads, wasted computation, and oversaturation of network bandwidth.

The Cortex data analysis toolkit further simplifies the extraction of passive data features as listed in Multimedia Appendix 1, with an example shown in Figure 7. Cortex provides prebuilt, parallelized and vectorized workflows in Python for PGHD extraction and featurization that operate across large data sets to generate interactive visualizations for the mindLAMP dashboard using the Vega visualization grammar (as listed in Table 2). It obviates the need to work directly with the LAMP protocol, allowing data scientists to reason about live actionable structured data entirely as data frames within their programming environment of choice. Through the vectorization of array operations and parallelization of function calls, Cortex is able to target high performance and cost-effectiveness, while maintaining data security and policy compliance. A sample execution plan for a particular analysis involving the GPS data is shown in Figure 8. The modular nature of PGHD captured by mindLAMP allows for personalization and creation of new digital biomarkers and analysis without the need for additional coding. Furthermore, the companion IDE manager abstracts away log-in and security issues by securely injecting an authenticated connection to the server into Cortex and the resulting analysis notebooks.

## Discussion

### Principal Findings

Research and clinical needs in digital medicine are evolving to use PGHD approaches to understand patient behavior and symptomatology [3]. To this end, by optimizing the system architecture for data throughput and substantial database write-loads, the LAMP platform supports high-performance data collection and real-time data analysis to enable, for example, larger machine learning models or just-in-time adaptive interventions that can leverage PGHD into actionable insights for patients and clinicians alike.

### Efficient Collection and Configuration

**Figure 9:**
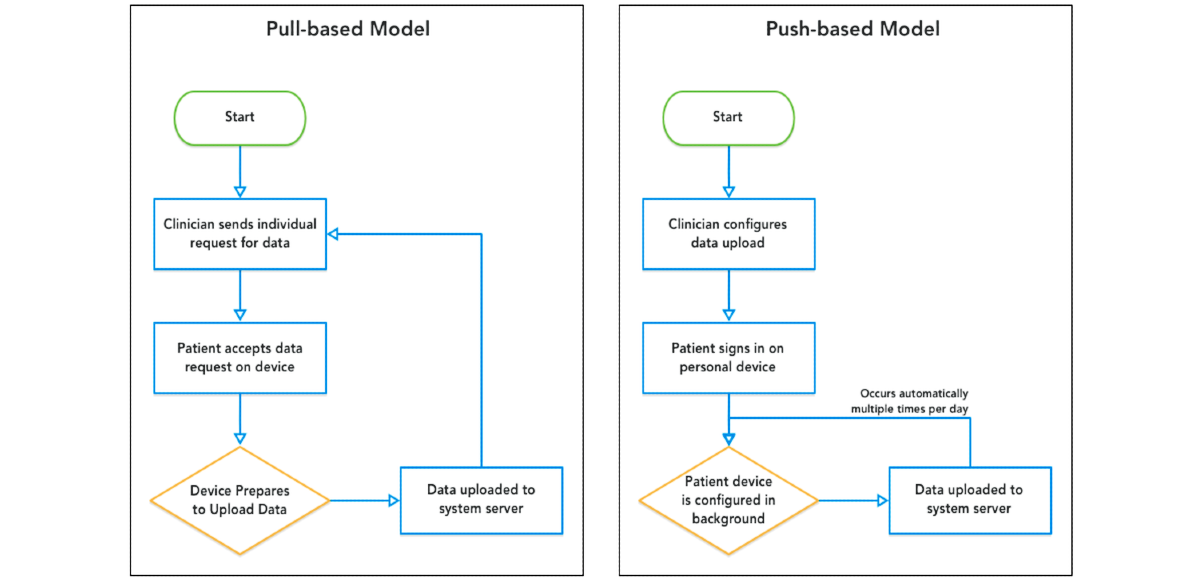
A pull-based model, in which clinicians must schedule data to be pulled from the device periodically versus a push-based model, in which clinicians preconfigure various sensors on the device to collect and push data to the server in real time.

Among the various approaches to data collection adopted in digital medicine, the *pull-based model* [13,14] shown in Figure 9, requires patients to activate a request and upload data from their mobile devices. This request can be scheduled and authorized. An example of the pull-based model is that during a clinical encounter, the clinician would use a portal to request data collection from the patient’s device for a period of 1 day; the patient would then have to approve this request in their smartphone app before data collection can begin. During the next clinical encounter, the clinician would be able to interpret the data in potentially meaningful ways.

**Figure 10:**
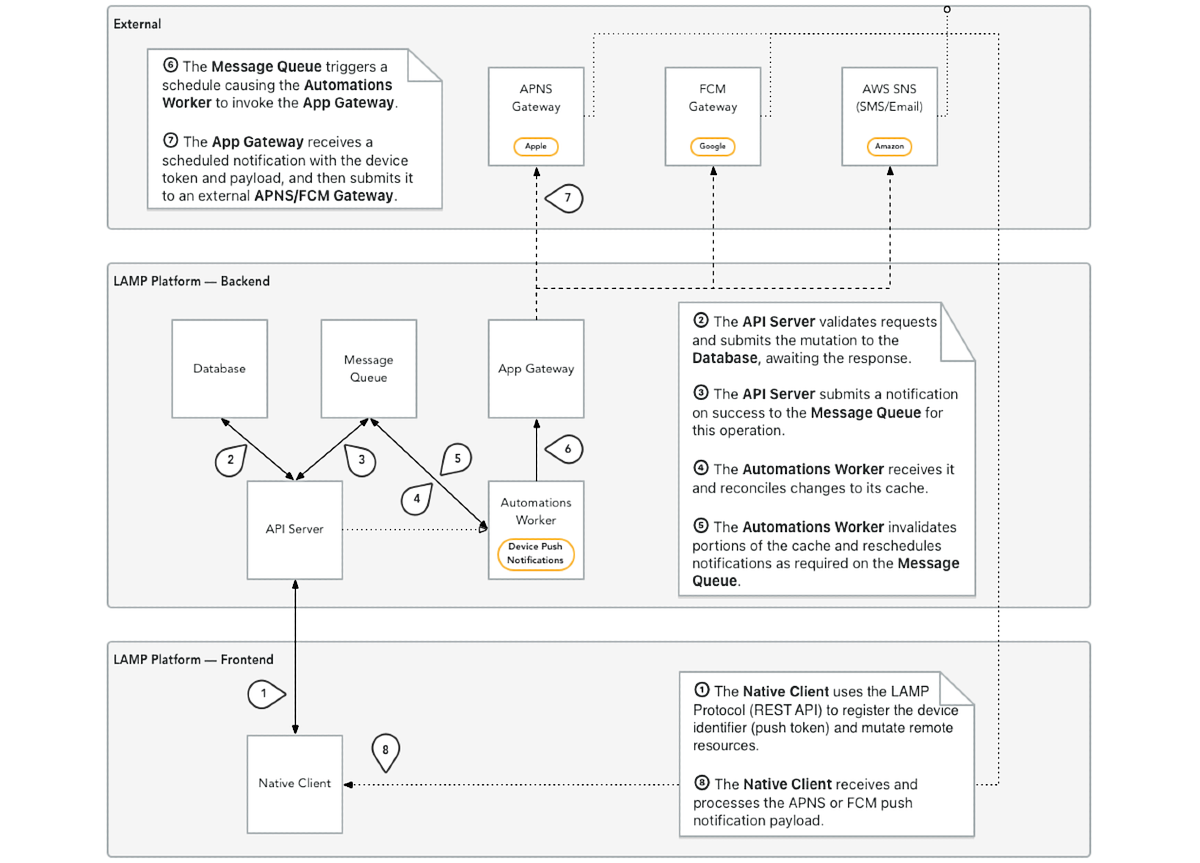
The detailed coordination required among the many components of the Learn, Assess, Manage, and Prevent platform involved in the submission of a push notification for a survey or gift card email for completion of a study; an example of the reporting of live intervention processing as made possible by a push-based model. Upon participant enrollment, survey delivery, gift card delivery, or intervention triggering, a message is pushed and logged to the Slack messaging service, a push-based model, to alert the research coordinator in real time. API: application programming interface; APNS: Apple Push Notification Service; FCM: Firebase Cloud Messaging; LAMP: Learn, Assess, Manage, and Prevent; REST: Representational State Transfer; SNS: Simple Notification Service.

**Figure 11:**
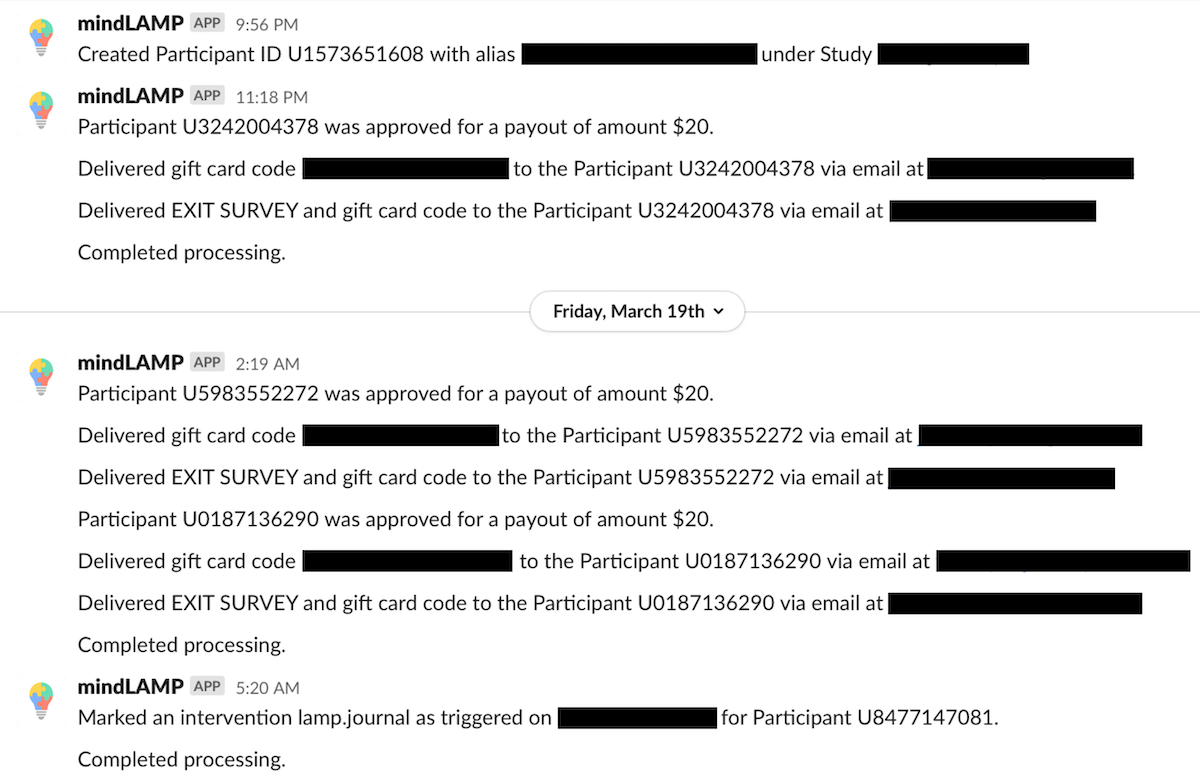
An example of the reporting of live intervention processing as made possible by a push-based model. Upon subject enrollment, survey delivery, gift card delivery, or intervention triggering, a message is pushed and logged to the Slack messaging service, also a push-based model, to alert the research coordinator in real time.

The LAMP platform, however, adopts a *push-based model* shown in Figure 9, where, in contrast, clinicians or research coordinators configure and schedule activities for patients to use and sensors from which measurements should be passively recorded ahead of time. The patients’ devices receive a configuration request that activates data collection in the background. As it is collected, the data are uploaded (*pushed*) to the back end periodically, available for processing and clinical insight ahead of time. This push-based approach reduces latency from collection of PGHD to the usability of that PGHD, for example, as real-time alerts in the context of research studies (Figures 10 and 11), or toward clinical decision-making with custom rules and alerts. It is important that clinicians or research coordinators communicate clearly and establish consent with the patient or subject about the various types of data being collected and the frequency of the data push.

The data collection processes are executed both actively during patient interactions with the mindLAMP app as well as passively while the app or mobile device is not in use. These data are securely uploaded to the organization’s backend systems and can be used immediately upon receipt for data analysis and logic to select interventions to display to the patient. Using push notifications sent to the mobile device, the platform promotes a high level of engagement with patients without explicitly requiring approval for data upload. Once a research study or clinic is configured, its documenting configuration can be exported and reimported by other LAMP-compatible systems or interfaces. This allows reproducibility in both clinics and research studies, for example, by attaching the configuration file to a research manuscript or clinical protocol.

### Consortium and Clinical Research Efforts

The LAMP platform is built and maintained collaboratively as an open platform to address the needs of many and integrate tools and resources to streamline workflows. In contrast to commercially available apps and services, the mindLAMP app may be used by organizations independently of our team through the deployment of a secure self-hosted backend. It can be customized and adapted without requiring specialized coding and deployment efforts, although others have also taken advantage of the extensibility of the platform to design and develop unique cognitive tests for their organization’s needs. Common data processing and analysis needs across clinical and research workflows are encapsulated by the platform and the Cortex data analysis toolkit to minimize the time between patient onboarding and affecting or assessing patient outcomes.

The LAMP platform is highly configurable to suit many needs across a broad range of both clinical and research use cases and strategies. Consortium partners are encouraged to share their use case and LAMP configurations. As shown in Textbox 1, there were many different potential configurations and use patterns across consortium members.

**Selected examples of configurations and use cases for the Learn, Assess, Manage, and Prevent platform across various consortium members.**

**Ecological momentary assessment tool**

- A total of 17 adults with substance use disorder who recently discharged from the hospital for this diagnosis completed daily assessments delivered via mindLAMP of mood, anxiety, sleep, social activity, and craving. No sensors were configured, and push notifications were enabled as reminders. Participants were able to view select survey responses in the Prevent tab. Results will be presented by the study team at the 34th Annual European College of Neuropsychopharmacology meeting in October 2021.

**Digital phenotyping+**

- A research study to examine circadian rhythms in bipolar disorder was conducted in which no activities were enabled or scheduled. The accelerometer, gyroscope, magnetometer, gravity, device motion, GPS, screen state, call and text, Bluetooth, and Wi-Fi sensors were enabled and configured to collect data at the highest possible frequency. Participants were able to view select sensor data in the Prevent tab.

**Both ecological momentary assessment and digital phenotyping+**

- A research study was conducted in which 100 college students were remotely enrolled to use the mindLAMP app for 1 month. Participants took 1 scheduled daily survey and 1 scheduled weekly survey, with provided optional tips and resources for managing stress, depression, and anxiety. The accelerometer, gyroscope, magnetometer, gravity, device motion, GPS, screen state, call and text, Bluetooth, and Wi-Fi sensors were enabled and configured to collect data at the highest possible frequency. Results are summarized in a paper published in 2021 [32].

**Individual patient study**

- A research study was conducted in which 50 participants with schizophrenia or bipolar disorder used the mindLAMP app for 1 year. Each participant was scheduled a daily standard battery of several surveys, the Jewels cognitive test, and the Spatial Span cognitive test. Optional tips and resources were provided, and the journaling, scratch card, and breathing exercise activities were made available for participants to use on their own volition. Participants were able to view their own data in the Prevent tab and worked with the research coordinator and psychiatrist to create custom surveys specific to individual participants’ needs. For example, 1 participant chose to create a water intake survey. Each participant’s notifications were scheduled individually by the research coordinator, instead of at a set time across all participants. The accelerometer, gyroscope, magnetometer, gravity, device motion, GPS, screen state, and call and text sensors were enabled and configured to collect data at the highest possible frequency.

**Clinical use**

- A clinical team in California offered dialectical behavioral therapy diary cards to all patients via mindLAMP. Before clinic visits and during their daily lives, patients would fill out these app-based dialectical behavioral therapy diary cards on their mobile device and were able to see their previous responses in the Prevent tab. No other activities were made available to the patients and no sensors were enabled for data collection. The diary cards were reviewed during each clinical session with the dialectical behavioral therapy therapist.

**Digital clinic**

- A clinic was established in which patients used the mindLAMP app with a new care team in addition to their ongoing care. Each patient was scheduled a daily standard battery of several surveys. Required tips and resources were provided along with required journaling, scratch card, and breathing exercise activities that were scheduled according to patient preferences. Patients were able to view their own data in the Prevent tab and worked with the digital navigator and clinician to create custom surveys or activities specific to individual patient’s needs. For example, 1 patient requested a set of self-management tips and resources. Each patient’s notifications were scheduled individually by the digital navigator. On the basis of each patient’s clinical goals each week, accelerometer, gyroscope, magnetometer, gravity, device motion, GPS, screen state, call and text, and other sensors were turned off or on with the goal of capturing relevant and actionable information to help manage care. A protocol for the clinic is published here [33].

**Intervention tool**

- A research team used the cognitive games in mindLAMP as a tool for cognitive remediation to offer attention and memory training to patients with clinical high risk for psychosis. A paper summarizing the results was published in 2021 [34]. The app was offered in the Mandarin Chinese language for this study.

To aid these joint research and clinical efforts, the LAMP consortium was founded to connect partners using the LAMP platform. The design and development of the platform occurs in an open-source, collaborative environment that many have taken advantage of to suggest features, report bugs, add documentation, and improve the overall quality and efficacy of the LAMP platform. Through its flexibility and interoperability, the platform encourages integration and cross talk between clinical and research contexts, and to this end, supports the implementation of a digital clinic [33] and the creation of a digital navigator role [35].

### Next Steps

The consortium further integrates directly into the development and feedback cycle using a community forum and bug tracking system, both available publicly. The community forum serves as a centralized resource for multiple teams or organizations to work with one another to assist with data analysis or troubleshooting and provide feedback about the LAMP platform. In addition, collaborators actively engage in making modifications to the source code (hosted through the public source code repository hosting service GitHub), make any suggested modifications or bug fixes, and then request that these changes be merged *upstream* into the distribution of the LAMP platform that is used by all. To learn more about the LAMP platform or help contribute, one can join the consortium or visit the open-source repository [36].

### Conclusions

Through the incorporation of patient- and clinician-centric feedback as well as a standards-based approach, the LAMP platform is designed to address important needs around the effective and compatible integration of PGHD into existing clinical systems for research and clinical care. It offers a flexible and comprehensive set of tools and solutions that can be configured and stitched together to function in a wide range of use cases, as used by members of the LAMP consortium. Its simplified programming interfaces are designed to securely handle a high throughput of PGHD as well as its companion metadata. With the integration of the Cortex data analysis toolkit, machine learning feature extraction, data processing, interactive visualization, and other essential tasks are simplified and coordinated seamlessly at low cost and high efficiency. In addressing technical challenges, the LAMP platform enables research and clinical teams to rapidly convert PGHD from widely accessible consumer smartphones and wearable devices into actionable clinical insights.

## Appendix

Multimedia Appendix 1 A full listing of Active, Passive, and Cortex data types currently supported by the Learn, Assess, Manage, and Prevent platform along with a description and expected components of the data.

## Abbreviations

API: application programming interface
FHIR: Fast Healthcare Interoperability Resources
IDE: integrated development environment
LAMP: Learn, Assess, Manage, and Prevent
PGHD: patient-generated health data
SMART: Specific, Measurable, Achievable, Realistic, and Timely
