# Supplementary material for: Enabling Research and Clinical Use of Patient-Generated Health Data (the mindLAMP Platform): Digital Phenotyping Study
Source: JMIR Mhealth Uhealth. 2022 Jan 7;10(1):e30557. doi: 10.2196/30557 (PMC8783287; doi:10.2196/30557)
Supplement: Multimedia Appendix 1 [file mhealth_v10i1e30557_app1.docx]

**Multimedia Appendix:** A full listing of Active, Passive, and Cortex data types currently supported by the Learn, Assess, Manage, and Prevent platform along with a description and expected components of the data.

| Data type | Category | Description |
| --- | --- | --- |
| Survey | Activity (assess) | Basic multi-question surveys with free text, multiple choice, and other response types. Data collection includes selected response as well as metadata, such as time taken per question. |
| Jewels | Activity (assess) | Adapted trails-making cognitive test (both A/B variants) where the user must tap on jewels/diamonds presented on screen in the specified order. Data collection includes tapped jewels and time taken between taps and between subsequent trials. |
| Box Game | Activity (assess) | Adapted spatial span cognitive test. Data collection includes tapped tile, correctness of tile tapped, time taken between taps and subsequent tiles. |
| Cats and Dogs | Activity (assess) | A novel cognitive assessment where several boxes appear on screen with a prompt indicating “cat” or “dog”—when the round begins, the user must tap all images matching the indicated prompt. Data collection includes tapped order, time between taps, and correctness of the tap. |
| Pop The Bubbles | Activity (assess) | The user is presented with a specific pattern of balloon colors to pop on the screen before the round begins, and during the round, the user most only pop those matching balloons and not any others. Data collection includes color of balloon popped, whether correct or not, and time taken for the current balloon to be tapped. |
| Balloon Risk | Activity (assess) | The user is presented with a balloon to be blown up several times to successively receive points until it pops at a randomly selected point. The user must decide whether to “cash out” or continue inflating the balloon. Data collection includes action taken, current points, time taken to decide, and whether the balloon was popped. |
| DBT^a^ diary card | Activity (assess) | A DBT^a^-specific diary card with questions. Data collection is customized to the set of emotions, target behaviors, and free-text responses along with the time taken to fill out the diary card. |
| Tips | Activity (learn) | Displays a set of rich text (including media) content. Data collection includes which content was accessed, how long the user spent on the content, and whether the user indicated the content was helpful or not. |
| Meditation | Activity (manage) | A breathing exercise appears with a configurable timer and optional configurable audio to play alongside the timer. Data collection includes time spent in meditation and whether the user indicated it was helpful or not. |
| Journal | Activity (manage) | A free-form text entry page that users share their thoughts and provide a sentiment of “good” or “bad” to mark the journal entry. Data collection includes the text entered by the user, the sentiment chosen, and time taken to type the text. |
| Scratch card | Activity (manage) | A photo selected by the user is displayed on the screen with a gray overlay that must be “scratched” off to reveal the photo. Data collection includes scratched pattern and time taken to scratch. |
| Device motion | Sensor | Collects background triaxial (x, y, and z) motion data from a device, such as linear accelerometer, gyroscope, gravity sensor, and magnetometer. |
| Pedometer | Sensor | Collects background step count. |
| GPS | Sensor | Collects background location information, such as latitude, longitude, altitude, and accuracy. |
| Nearby devices | Sensor | Scans Wi-Fi and Bluetooth devices in the background, collecting hashed signal name and signal strength. Can be used to assess social density and proximity to other devices. |
| Call and text | Sensor | Collects information about incoming and outgoing calls and texts, including the encrypted duration of a call or the length of a text message. Note, no phone number or content of the message is captured. |
| Sleep | Sensor | Collects information on device-predicted sleep onset and wake onset. |
| Heart rate | Sensor | On wearable devices, collects heart rate measurements and heart rate variability periodically in the background. |
| Screen state | Sensor | Collects information about number of notifications received per day and total duration of device activity. Note, no actual screen data is recorded. |
| Nutrition | Sensor | On devices with Apple HealthKit or Google Fit configured to use a companion nutrition tracking app, nutrition data is also imported per day. |
| Blood glucose | Sensor | On devices with a paired smart blood glucose monitor, measurements are imported. |
| Blood pressure | Sensor | On devices with a paired smart blood pressure monitor, measurements are imported. |
| Oxygen saturation | Sensor | On devices with a paired smart oxygen saturation monitor, measurements are imported. |
| Body temperature | Sensor | On devices with a paired smart body thermometer, measurements are imported. |
| Respiratory rate | Sensor | On devices with a paired smart respirometer, measurements are imported. |
| Activity recognition | Sensor | On supported devices, collects background data and estimates the current potential activity being performed by the user, such as walking, biking, and driving. |
| Screen activity | Cortex | Computes a list of bouts (on-off) of screen activity from accelerometer and screen state data. |
| Significant locations | Cortex | Computes the coordinates and radii of significant locations visited during a specified time window. |
| Sleep periods | Cortex | Computes the expected sleep periods for a set of accelerometer data spanning a 1-day window. |
| Survey results | Cortex | Scores surveys dynamically reported in terms latency of response and other meta data from survey question categories. |
| Number of trips | Cortex | Label GPS readings as being either “stationary” or “transitionary” and tabulate the duration of time spent per day in each state. Note, the location of trips is not recorded. |
| Distance traveled | Cortex | Translates trips to a combination of distance and duration traveled per day. Note, the location of travel is not recorded. |
| Home time | Cortex | Determines the amount of time spent at home (computed from significant locations) in a given 1-day window. Note, the actual location is not recorded. |
| Green space | Cortex | Determines the quality of “green space,” or trees/parks near the GPS points in a given time window. Note, actual locations are not recorded. |
| Entropy | Cortex | Detects degree of variance in daily mobility patterns through the time window. |
| Activity segments | Cortex | Synthesizes daily segments of mobility from other secondary features. |
| Call degree | Cortex | How many persons contacted and for how long. Note, no phone number or content of the message is captured. |
| Charging frequency | Cortex | Determines how frequently and for how long the device is charged per day. |
| Notification checks | Cortex | Uses screen state data to discern notification pickups and counts the number of pickups per day. |
| Beta values | Cortex | Extracts meaningful metrics related to effort and process of cognition from cognitive test results. |

^a^DBT: dialectical behavioral therapy.
